# Delayed Acute Coronary Syndrome Caused by Multiple Bee Stings: A Rare Case of Kounis Syndrome

**DOI:** 10.7759/cureus.14120

**Published:** 2021-03-26

**Authors:** Apostolos Dimos, Andrew Xanthopoulos, Dimitrios Bismpos, Filippos Triposkiadis, John Skoularigis

**Affiliations:** 1 Department of Cardiology, University Hospital of Larissa, Larissa, GRC; 2 Department of Cardiology, Interbalkan Medical Center, Thessaloniki, GRC

**Keywords:** kounis syndrome, bees, acute coronary syndrome, troponin, antiplatelet

## Abstract

A 51-year-old female patient was admitted to our hospital for medical evaluation and treatment of a syncopal episode following multiple bee stings. The syncopal episode was attributed to an allergic reaction and the patient was treated with intravenous hydration and anti-histamines. Twenty-four hours later, the patient manifested an acute coronary syndrome with chest discomfort, electrocardiographic disorders, and myocardial enzyme motility (including troponin). Coronary angiography was performed without revealing pathological findings and she was diagnosed with Kounis syndrome type I. The management of the patient included administration of single antiplatelet therapy combined with a calcium channel blocker (CCB). The patient follow-up was uncomplicated. In patients with Kounis syndrome type I undergoing a normal coronary angiography, in the absence of specific guidelines, single antiplatelet therapy and CCB may be a reasonable approach.

## Introduction

Kounis syndrome (KS) is a potentially life-threatening medical emergency that includes both a severe allergic reaction and an acute coronary syndrome (ACS) [1,2]. In the only prospective study undertaken so far the incidence of Kounis syndrome at the emergency department in one year among all admissions and allergy patients was estimated to be 19.4 per 100,000 (27/138,911) and 3.4% (27/793), respectively [3], while the prevalence of KS in the United States among patients hospitalized for allergic reactions has been estimated to be 1.1% [4]. Generally, the prognosis of KS is good. Serious complications are rare, i.e., cardiogenic shock occurs in 2.3%, cardiac arrest occurs in 6.3% and the death rate is 2.9% among KS patients while the mortality rate is similar in both genders (3.0% in males and 2.2% in females) [1,4]. KS is mostly described through case reports since currently, specific treatment guidelines are lacking. Herein, we report a case of a 51-year-old woman presenting with a delayed ACS caused by hundreds of bee stings and her management.

## Case presentation

**Figure 1:**
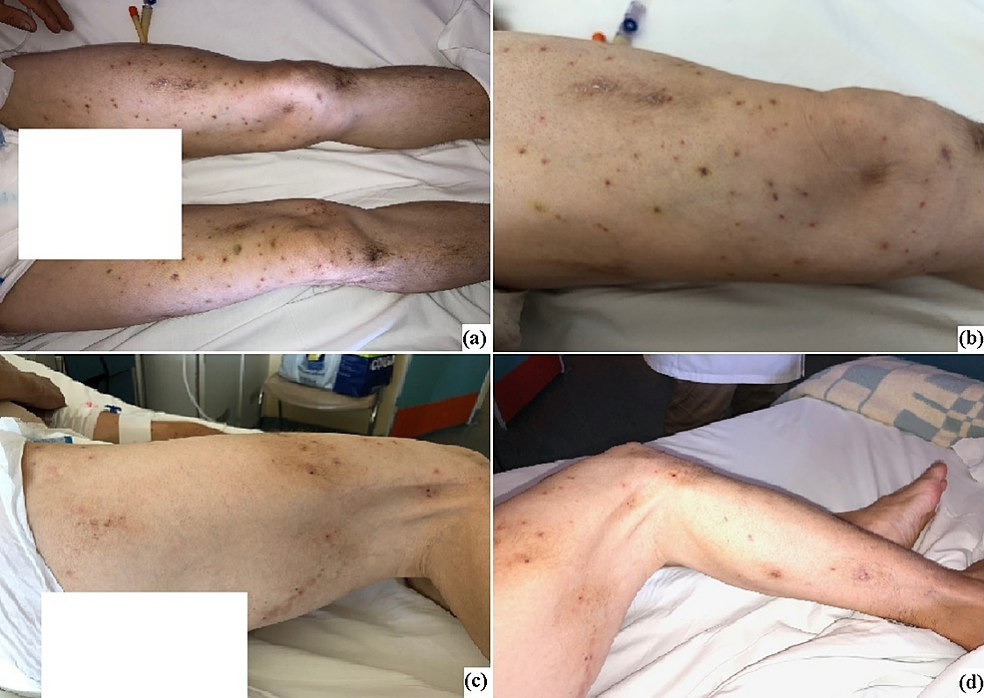
Multiple bees stings in the patient's lower extremities.

A 51-year-old female patient with a history of heterozygous beta-thalassemia and hyperthyroidism was transferred to the ED due to a syncopal episode and sleepiness after an allergic bee reaction. Before admission to the ED, the patient was found on the ground hemodynamically stable (blood pressure {BP} = 110/90mmHg, heart rate {HR} = 95bpm), breathing normally without the need for supplementary oxygen delivery, with multiple dermal damages in her face, body, and limps that were compatible with insect stings (bees) (Figure 1) and received intravenously methylprednisolone and antihistamines during her attendance at the ER. At the first medical examination in ED, she had a low-grade fever (T = 37.8°C) and she continued to be hemodynamically stable (BP = 120/80mmHg, HR = 90bpm) with no need for oxygen (peripheral capillary oxygen saturation {SaO2} = 98%). S1 and S2 were rhythmic without additional sounds, with bilateral palpable peripheral pulses, and without peripheral lower limb edema. The patient reported no allergies from her medical history.

**Figure 2:**
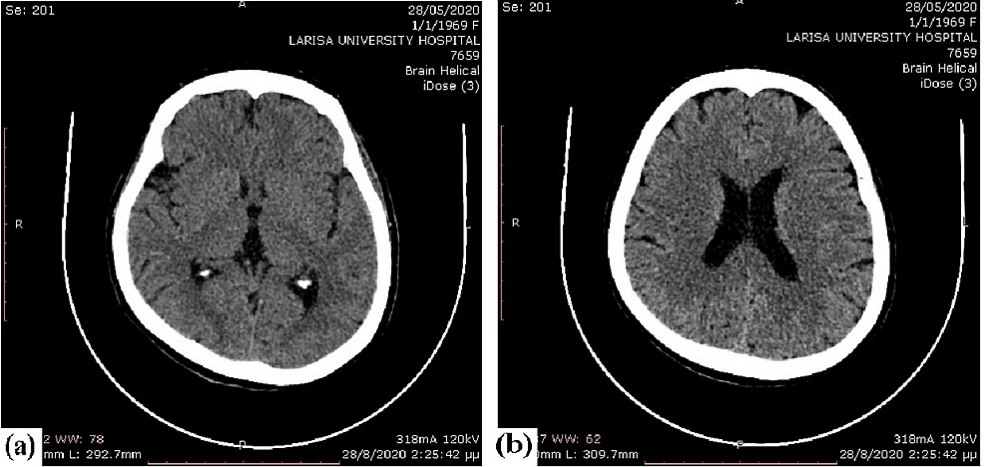
CT brain of the patient revealed no pathological findings.

All the stings were removed and skin damages were pampered. Afterward, because of the fall on the ground and the sleepiness, the patient underwent a CT brain scan which revealed no pathological findings (Figure 2). The patient was admitted to the Internal Medicine Department for further investigation and treatment.

Initial laboratory tests showed prolongation of prothrombin time (PT), international normalized ratio (INR), and activated partial thromboplastin time (aPTT), microscopic hematuria, and increased muscle enzymes. The patient was treated with intravenous hydration and anti-histamines per os, while intravenous antibiotic therapy with ampicillin/sulbactam was added due to infection of certain affected by stings skin lesions with the simultaneous presence of fever and increased C-reactive protein (CRP) value. The ranges of PT, INR, and aPTT were improved during the first day of hospitalization without any evidence of hemorrhage. The patient underwent lumbar puncture due to a disturbing level of consciousness (sleepiness with Glasgow Coma Scale value 13) and a central nervous system infection was ruled out (0 WBC and normal levels of albumin cerebrospinal fluid sugar). Coronavirus disease-19 (COVID-19) infection was ruled out after a negative polymerase chain reaction (PCR) test of the pharyngeal swab.

**Figure 3:**
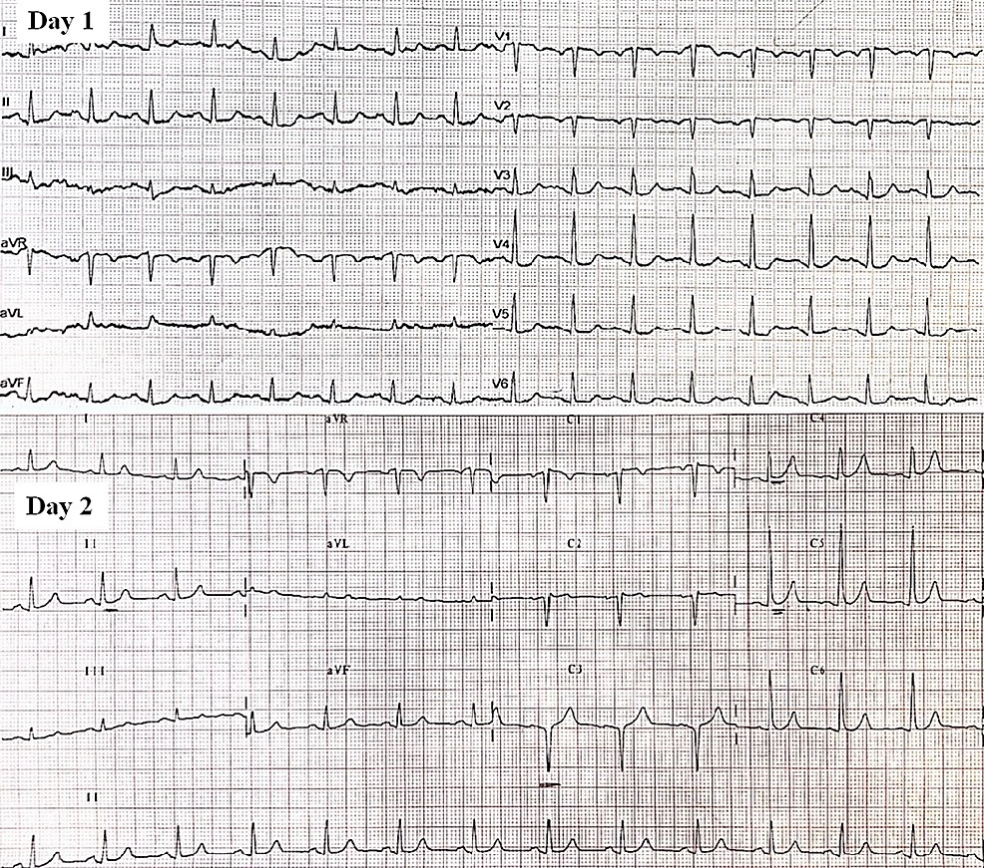
The ECG of the patient demonstrating repolarization abnormalities with cup-shaped morphology of ST interval and giant positive T waves at the precordial leads. ECG: electrocardiogram

On the second day of hospitalization, the patient complained of tight chest pain lasting up to two hours which was accompanied by an increase of myocardial enzymes (including troponin) levels. Table 1 and Table 2 list the main daily laboratory values including arterial blood gases measurements. The electrocardiogram (ECG) showed repolarization abnormalities with cup-shaped morphology of ST interval and giant positive T waves at the precordial leads (Figure 3). An ACS was diagnosed and the patient was transferred to the Cardiology Clinic for further evaluation and treatment.

**Table 1:** Main daily laboratory values. WBC: white blood cell; Hb: hemoglobin; Hct: hematocrit;  PLT: platelet; K+: potassium ion; Na+: sodium ion; CPK: creatine phosphokinase; SGOT: serum glutamic oxaloacetic transaminase; LDH: lactate dehydrogenase; CRP: C-reactive protein; PT: prothrombin time; aPTT: activated partial thromboplastin time; INR: international normalized ratio

	Day 1	Day 2	Day 3	Day 4	Day 5
WBC (/uL)	17800	20600	11700	6900	5600
Hb (g/dL)	11.6	10.9	10.4	11.2	11.4
Hct (%)	37.5	34.3	33.1	35.5	35.2
PLT (/uL)	246000	297000	290000	330000	324000
K^+^ (mmol/L)	4.2	3.6	4.45	4.3	4.1
Na^+^ (mmol/L)	139	143	142	141	142
CPK (U/L)	375	2676	4679	2712	908
Troponin (μg/L)	-	0.026	0.015	0.010	0.010
SGOT (IU/L)	72	149	246	221	125
LDH (IU/L)	621	608	614	514	413
CRP (mg/dL)	0.1	1.7	0.38	0.17	0.1
PT(sec)	17.4	12.7	12.5	12.5	12.5
aPTT(sec)	96.5	22.3	22.6	23	23
INR	1.5	1.09	1.02	1.07	1.02

**Table 2:** Arterial blood gases measurements. pH: potential of hydrogen; pO2: partial pressure of oxygen; pCO2: partial pressure of carbon dioxide; HCO3: bicarbonate; SatO­2: peripheral capillary oxygen saturation; FiO2: fraction of inspired oxygen

Day 1		Day 3	
pH	7.45	pH	7.42
pO_2_	84mmHg	pO_2_	80mmHg
pCO_2_	37mmHg	pCO_2_	36mmHg
HCO_3_	25mmol/L	HCO_3_	23.4mmol/L
Lactate	1.1mmol/L	Lactate	1.0mmol/L
SatO_2_	98%	SatO_2_	97%
FiO_2_	21%	FiO_2_	21%

**Figure 4:**
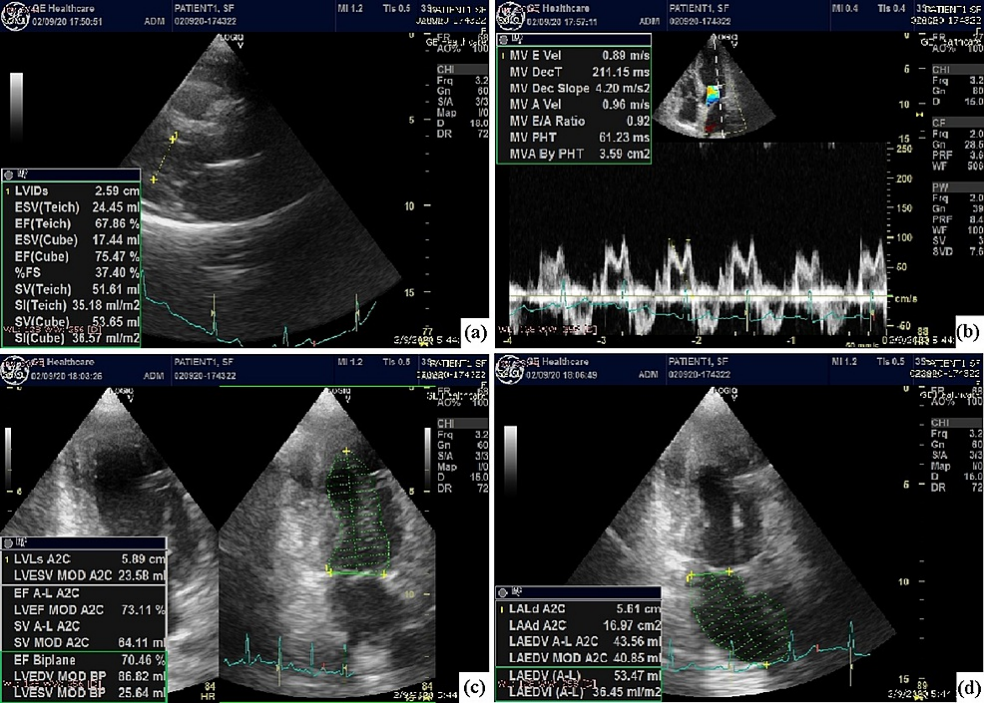
Echocardiographic evaluation of the patient. (a) Parasternal long axis. Normal dimensions of the left atrial and ventricular chambers; (b) transmitral E to A ratio (E≈A);  (c and d)  apical 4 and 2 chambers view. The normal systolic function of the left ventricle (Simson method).

**Figure 5:**
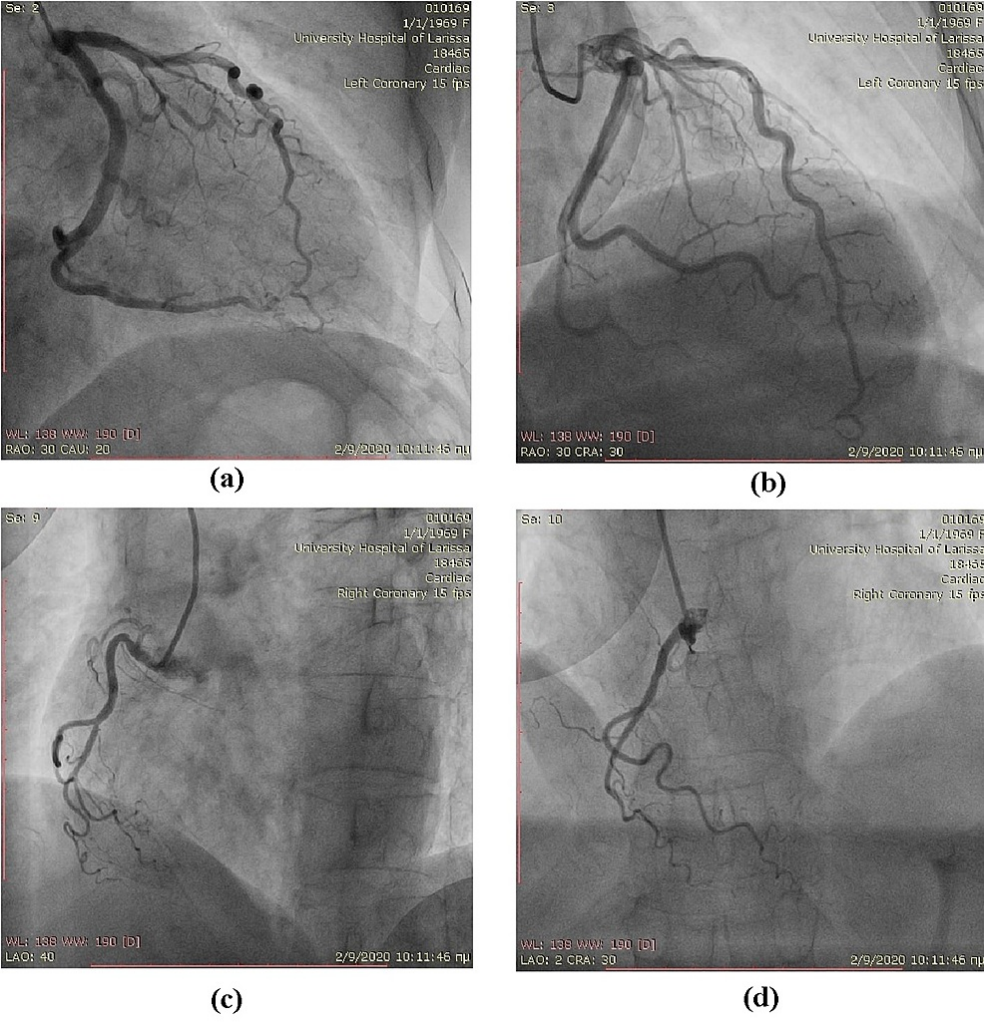
Coronary catheterization revealing normal coronary arteries.

A detailed echocardiographic analysis revealed no left ventricular wall motion abnormalities (Figure 4). Coronary angiography was performed on the fifth day of the patient’s hospitalization from which no coronary artery stenosis was found (Figure 5). As the patient completed six days of hospitalization with stabilization of the clinical picture and normalization of myocardial enzymes, she was discharged with instruction for a single antiplatelet therapy (clopidogrel) combined with a calcium channel blocker.

 

On the re-examination of the patient 30 days later she was asymptomatic with regression of the skin lesions and normalization of the ECG. The ambulatory electrocardiographic recording (HOLTER) examination did not reveal any arrhythmias or conduction disturbances and she was advised to be followed-up regularly.

## Discussion

Kounis syndrome is a hypersensitivity coronary disorder evolving into ACS, induced by exposures to drugs, food, environmental and other triggers [1]. Back in 1991, Kounis and Zavras were the first to describe the “allergic angina syndrome” as a coronary spasm progressed to allergic acute myocardial infarction [2].

Although KS can be encountered at any age, the most commonly affected age group is 40-70 years old (68%), with a history of previous allergy, hypertension, smoking, diabetes, and dyslipidemia being considered significant risk factors. The most common triggers of KS are antibiotics (27.4%) followed by insect bites (23.4%), whereas 80% of the cases occur within the first hour of exposure to the trigger [1]. This syndrome is divided into three groups (I, II, III). In the type, I variant (the most common variant, 72.6%), also known as allergic vasospastic angina, the release of immune response mediators induces coronary artery spasm. This may lead to myocardial infarction with non-obstructive coronary arteries (MINOCA) with an increase of cardiac enzymes, including troponin. In the type II variant (22.3%), the release of the same mediators induces coronary artery spasm and sometimes plaque erosion or rupture manifesting as acute myocardial infarction, in people with underlying asymptomatic coronary artery disease. Type III variant (5.1%) includes patients with coronary artery stent thrombosis caused by an allergic reaction [5].

KS seems to be an infrequently diagnosed clinical entity. The main inflammatory cells that are involved in the KS progress are mast cells interacting with macrophages and T-lymphocytes via multidirectional stimuli [5]. In 2015, Lippi and colleagues found that in patients who were admitted to the emergency department suffering from anaphylaxis, angioedema, urticaria, and urticaria-angioedema, the troponin I levels were significantly increased in comparison with healthy controls. This denotes that the heart and especially the coronary arteries constitute primary targets of anaphylaxis [6].

Allergic inflammation goes through three phases, the early phase which lasts minutes, the late phase which lasts from two hours to two days, and the chronic phase which follows a continuous, persistent, and repetitive allergen exposure and lasts as long as the allergen is present. Early-phase reactions (or type I immediate hypersensitivity reactions) occur in sensitized individuals within minutes of allergen exposure and are mainly related to the secretion of specific mast cell cytokines. At late-phase reactions mast cells, responding to immunoglobulin E (IgE) and allergen, produce new cytokines, chemokines, and proliferating mediators at a slower rate. When allergen exposure is continuous or repetitive, inflammation persists, and many immune cells with modified activity migrate and settle in the areas affected by the allergen (chronic allergic inflammation) [7].

In our case, the initial symptom of the patient was syncope and sleepiness. Tight chest pain and electrocardiographic changes with upward movement of myocardial enzymes (including troponin) were delayed for 24 hours. Therefore, a novelty of the present case is that the patient suffered from an allergic reaction accompanied by a delayed ACS.

Until now, guidelines for the treatment of KS are lacking and all available data on the effectiveness or not of treatment approaches were extracted from individual case reports or case series. Therefore, cardiologic management of KS patients is challenging, while management of anaphylaxis should follow the anaphylaxis guidelines [8].

The utility of common medications used in ACS, such as antithrombotic drugs, nitrates, and b-blockers in patients with KS is unknown given the potential risk of aggravating an ongoing anaphylactic reaction [9]. Although aspirin has important anti-platelet and anti-inflammatory effects in patients with coronary artery disease and ACS, it may also be the cause of an allergic reaction and may subsequently lead to anaphylaxis [10]. The use of beta-blockers is extremely beneficial in the treatment of ACS but these drugs may offset some of the beneficial effects of epinephrine which is the cornerstone of the treatment of anaphylaxis [11]. In that case, glucagon may be used as an antidote in patients with anaphylaxis and hypotension who are already on beta-blockers or received during the management of the ACS [12]. Although calcium channel blockers are not the first line of therapy in ACS, they may be considered as an initial anti-ischemic drug along with nitrates since the vasospasm is the primary mechanism of the ACS in the KS [13].

Airway, breathing, circulation, and level of consciousness should be immediately assessed at the acute management of a patient with KS. Epinephrine is a lifesaving medication in anaphylaxis. Although there was concern that the use of adrenaline in anaphylaxis could induce arrhythmias and ACS, the benefit of the usual administered dose outweigh the risk [13,14]. Glucocorticoids when administered early after coronary occlusion may interfere with myocardial scar formation. Cases of vasospastic angina have been reposted after corticosteroids use, a condition that may worsen pre-existing ischemia [15]. A longer duration of corticosteroid administration could be the cause of cardiovascular events like myocardial infarction through obesity, insulin resistance, glucose intolerance, dyslipidemia, hypertension, and direct effect on coronary vessels. Perivascular inflammation, myocardial fibrosis, and wall thinning leading to cardiac aneurysms and cardiac wall rupture may be the consequences of impaired wound healing and scar formation after the use of corticosteroids [16,17].

The patient was initially treated for the allergic reaction to bee stings with corticosteroids (once in the first contact) and antihistamines (first 24 hours) whereas a single antiplatelet treatment (clopidogrel instead of aspirin to avoid an allergic exacerbation) combined with a calcium channel blocker were chosen after the diagnosis of ACS. Thirty days after her discharge the patient was totally asymptomatic fully functional and a follow-up was scheduled regularly.

## Conclusions

The diagnosis and management of KS can be challenging in specific cases such as the one reported herein. Common accepted diagnostic and treatment algorithms are lacking. In the present case, the patient was treated with single antiplatelet therapy combined with CCB.

## References

[1] Abdelghany M, Subedi R, Shah S, Kozman H (2017). Kounis syndrome: a review article on epidemiology, diagnostic findings, management and complications of allergic acute coronary syndrome. Int J Cardiol.

[2] Kounis NG, Zavras GM (1991). Histamine-induced coronary artery spasm: the concept of allergic angina. Br J Clin Pract.

[3] Akoz A, Tanboga H, Emet M, Bayramoglu A, Kizrak Y, Kantarci M, Aslan S (2013). A prospective study of Kounis syndrome: clinical experience and cardiac magnetic resonance imaging findings for 21 patients. Acta Med Mediterr.

[4] Desai R, Parekh T, Patel U (2019). Epidemiology of acute coronary syndrome co-existent with allergic/hypersensitivity/anaphylactic reactions (Kounis syndrome) in the United States: a nationwide inpatient analysis. Int J Cardiol.

[5] Kounis NG (2016). Kounis syndrome: an update on epidemiology, pathogenesis, diagnosis and therapeutic management. Clin Chem Lab Med.

[6] Lippi G, Buonocore R, Schirosa F, Cervellin G (2015). Cardiac troponin I is increased in patients admitted to the emergency department with severe allergic reactions. A case-control study. Int J Cardiol.

[7] Galli SJ, Tsai M, Piliponsky AM (2008). The development of allergic inflammation. Nature.

[8] Fassio F, Losappio L, Antolin-Amerigo D (2016). Kounis syndrome: a concise review with focus on management. Eur J Intern Med.

[9] Biteker M (2010). Current understanding of Kounis syndrome. Expert Rev Clin Immunol.

[10] Kowalski ML, Asero R, Bavbek S (2013). Classification and practical approach to the diagnosis and management of hypersensitivity to nonsteroidal anti-inflammatory drugs. Allergy.

[11] White JL, Greger KC, Lee S, Kahoud RJ, Li JT, Lohse CM, Campbell RL (2018). Patients taking β-blockers do not require increased doses of epinephrine for anaphylaxis. J Allergy Clin Immunol Pract.

[12] Thomas M, Crawford I (2005). Best evidence topic report. Glucagon infusion in refractory anaphylactic shock in patients on beta-blockers. Emerg Med J.

[13] Leow SN, Tang WS (2019). Angina after anaphylaxis treatment. Malays Fam Physician.

[14] Kounis NG, Koniari I, Tsigkas G, Soufras GD, Plotas P, Davlouros P, Hahalis G (2020). Angina following anaphylaxis: Kounis syndrome or adrenaline effect?. Malays Fam Physician.

[15] Okumura W, Nakajima M, Tateno R, Fukuda N, Kurabayashi M (2014). Three cases of vasospastic angina that developed following the initiation of corticosteroid therapy. Intern Med.

[16] Walker BR (2007). Glucocorticoids and cardiovascular disease. Eur J Endocrinol.

[17] Shokr M, Rashed A, Lata K, Kondur A (2016). Dexamethasone associated ST elevation myocardial infarction four days after an unremarkable coronary angiogram-another reason for cautious use of steroids: a case report and review of the literature. Case Rep Cardiol.

